# Classifying Convergences in the Light of Horizontal Gene Transfer: Epaktovars and Xenotypes

**DOI:** 10.1093/molbev/msaf279

**Published:** 2025-10-28

**Authors:** James O McInerney

**Affiliations:** Department of Evolution, Ecology and Behaviour, University of Liverpool, Liverpool L69 3BX, United Kingdom

## Abstract

The classification of living systems presents significant challenges due to the prevalence of gene transfer between genomes. Traditional taxonomic systems have been designed to describe tree-like evolution and consequently struggle to accommodate network-like evolutionary patterns. In this perspective, I consolidate and clarify terminology for describing organisms whose evolutionary history has not been strictly tree-like. I introduce two complementary concepts: **epaktovars**, groups (≥2) of organisms exhibiting convergent phenotypes through independent acquisition of similar functions, whether via horizontal gene transfer (HGT) or independent evolution of analogous solutions, and **xenotypes**, organisms that share homologous genes acquired through HGT, regardless of whether these shared genes produce similar or different phenotypes. The epaktovar concept mirrors the previously established concept of epaktologs (independent assembly of similar protein domain architectures), while xenotypes extends the concept of xenologs (horizontally transferred homologous genes) to the genome level. Recent research on homoplastic patterns in pangenome evolution enhances our understanding of these phenomena. These concepts also have important applications in synthetic biology and de-extinction efforts, where genetically modified organisms and reconstructed extinct species can be understood as xenotypes and epaktovars of their genetic donors, providing a framework for classifying organisms whose genetic composition has been shaped by human intervention rather than natural evolutionary processes. These terms collectively provide a framework for describing both phenotypic convergence arising through any evolutionary mechanism and shared genetic material resulting specifically from gene transfer across diverse lineages.

## Introduction

The Linnean binomial classification system has served as the foundational taxonomic system for almost 300 years (Linnaeus 1758). This approach functions perfectly in a world of strictly tree-like evolution but faces fundamental challenges when applied to genomes with a history of recombination (Bapteste et al. 2012). While Darwin's “descent with modification” describes vertical inheritance patterns, prokaryotes in particular—though it is common also in eukaryotes (McInerney 2017)—engage in horizontal gene transfer (HGT), hybridization, and recombination in its various forms, complicating their evolutionary histories.

Over the last century or so, evolutionary biology has developed specific terminology to describe gene relationships based on their history. *Homologs* are genes that share a common ancestor, regardless of the evolutionary process that led to their current state. This broad category is further refined into *orthologs* (homologs that diverged following a speciation event), *paralogs* (homologs that diverged following gene duplication within a lineage), and *xenologs* (homologs acquired through HGT from a different lineage) (Fitch 1970; Gray and Fitch 1983). There are additional terms, such as ohnologs (homeologs), which describe paralogs that have arisen because of whole genome duplication (Xie et al. 2016), though this is a distinction that refers to the mechanism of duplication, rather than being a separate class of homolog. While these terms effectively describe gene-level relationships, they become inadequate when addressing genome-level phenomena resulting from extensive introgressive gene transfer.

Comparative genomic analysis has shown that within a closely related group of organisms, there is usually a relatively stable “core” component of genetic material and variable “accessory” genetic parts, and this has led to the description of the pangenome phenomenon (Tettelin et al. 2005). The sequencing of three *Escherichia coli* strains demonstrated that they shared less than 40% of their gene content, with the remainder being unique to one or two genomes (Welch et al. 2002). This variation was shown to arise primarily from horizontal acquisition of genes rather than differential loss from a gene-rich ancestor. The pangenome is a term that describes the totality of gene families found in a species (McInerney et al. 2017; Domingo-Sananes and McInerney 2021; McInerney 2022). Pangenomes can be remarkably large (Bobay and Ochman 2017; Her and Wu 2018), particularly in prokaryotes, with the accessory genome typically being acquired through HGT. Although the pangenome concept was originally described in prokaryotes, eukaryotic genomes also experience significant HGT, hybridization, introgression, and transposable element activity (Keeling and Palmer 2008; Soucy et al. 2015). Eukaryotes possess additional complexities including sexual reproduction, meiosis, polyploidization, and extensive genome restructuring that create unique patterns of gene transfer and convergent evolution (Moran and Jarvik 2010).

Pangenomes are a fundamental part of the evolutionary history of life on the planet. Importantly, many genes are repeatedly acquired across divergent lineages, resulting in distantly related organisms becoming genetically similar for specific genomic regions. While xenologs describe individual genes acquired through horizontal transfer, we lack terminology to describe organisms that share one or multiple xenologs, potentially conferring similar phenotypes despite different evolutionary histories. Similarly, current terminology inadequately addresses cases where organisms independently evolve similar phenotypes through different genetic mechanisms (analogous traits) or through acquisition of similar gene sets from different sources (homologous traits). These phenomena are increasingly recognized as fundamental patterns in evolution rather than exceptional cases.

**Fig. 1.**
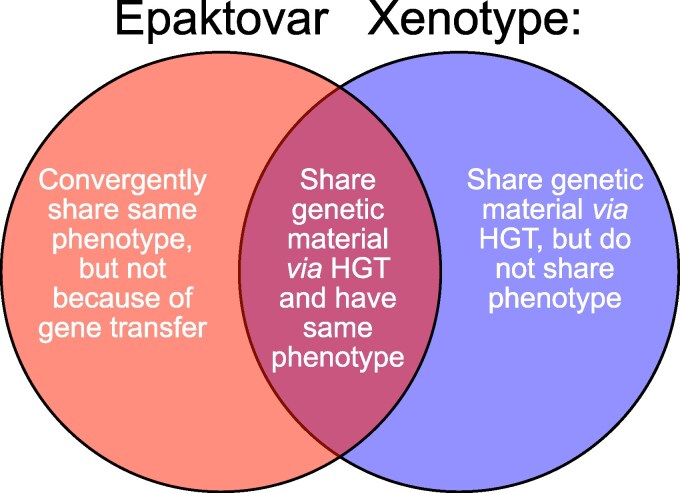
Clarification of the relationships between epaktovars and xenotypes.

I therefore propose two new terms—“Epaktovars” and “Xenotypes”—to fill the gap in evolutionary terminology. “Epaktovars” (from Greek “epaktos” (ἐπακτός), meaning “brought in” and “var” indicating variety) describes organisms that have independently acquired similar phenotypes, mirroring the concept of “epaktologs” at the protein level (Nagy et al. 2011). “Xenotypes” describes organisms that share genetic material that has been acquired through horizontal transfer, regardless of quantity, mechanism of transfer (artificial or natural), or phenotypic effect (e.g. transfer of a promoter region might produce a phenotype i.e. completely unrelated to the phenotype conferred by the promoter in the donor organism). The term “Xenotype” derives from the Greek “xenos” (ξένος) meaning foreign and “typos” (type), emphasizing the foreign origin of the shared genetic material. The concept of xenotypes complements epaktovars by focusing specifically on the shared genetic material rather than phenotypic similarity, and it extends the concept of xenologs (Gray and Fitch 1983) to the organismal level. Figure 1 outlines the distinction, and need for, the two terms.

While epaktovars focus on acquired phenotypic similarity regardless of the underlying genetic mechanisms, xenotypes focus on acquired genetic similarity regardless of the resulting phenotypes. This terminology is necessary because traditional concepts fail to capture two distinct evolutionary phenomena: phenotypic convergence arising through independent evolution and genetic convergence arising through horizontal transfer. For example, two distantly related organisms that acquire the same mobile genetic element become xenotypes with respect to that element, and if that element produces similar traits or phenotypes in both organisms, they also become epaktovars, despite their disparate evolutionary histories. Conversely, two organisms that independently evolve similar phenotypes through entirely different genetic mechanisms become epaktovars but not xenotypes. The suffixes in these terms also align with established conventions in biology and genetics. For “epaktovar,” the suffix “var” deliberately follows the precedent set by terms like “serovar,” “biovar,” and “cultivar,” which are used to distinguish variants within a species based on biological properties. Similarly, “xenotype” employs the suffix “type” to situate it within the family of biological classification terms like “genotype,” “phenotype,” and “ecotype,” emphasizing its role in describing a fundamental aspect of biological identity that emerges from genetic relationships. However, unlike “phenotype,” which describes observable traits, or “ecotype,” which describes ecological adaptation, “xenotype” specifically addresses the genetic basis of the kinship created through HGT. These terminological decisions create linguistic coherence with existing taxonomic and genetic vocabulary while introducing necessary concepts to describe the network-like patterns observed in prokaryotic evolution.

Together these terms offer several benefits to the research community. First, they provide precise language for describing convergent genomic patterns, facilitating clearer communication about evolutionary relationships. Second, they enable more accurate classification of organisms whose relationships cannot be fully captured by traditional phylogenetic approaches. Third, they create a framework for studying the deterministic versus stochastic aspects of genome evolution (e.g. Beavan et al. 2024). Finally, they bridge molecular and phenotypic perspectives by connecting genetic acquisition to functional outcomes.

### Mechanisms of Epaktovar and Xenotype Formation

Pangenomes can be viewed as ecosystems, with individual genomes acting as evolving localities (McInerney 2022). This perspective highlights how epaktovars can emerge through both deterministic and stochastic processes. Similar ecological “rules” strongly influence gene acquisition and retention across diverse lineages, leading to convergent genome compositions (Dorrell et al. 2023). Even with the dynamic nature of HGT, predictable patterns govern which genes are acquired and retained together (Whelan et al. 2020). At least three primary types of gene–gene relationships shape pangenome structure and contribute to xenotype and epaktovar formation.

Eukaryotes employ several mechanisms that parallel prokaryotic HGT while introducing additional routes to xenotypic and epaktovar relationships. Interspecies hybridization, particularly common in plants and some animals, can introduce thousands of genes from distantly related species in a single event (Abbott et al. 2013). Endosymbiotic gene transfer, where genes from mitochondria, chloroplasts, or endosymbionts are incorporated into the host nuclear genome, has also played a key role in eukaryotic evolution (Timmis et al. 2004).

Several mechanisms contribute to the formation of xenotypes. First, HGT allows genetic material to move between organisms *via* transformation (Avery et al. 1944), conjugation (Lederberg and Tatum 1946), transduction (Zinder and Lederberg 1952), and Gene Transfer Agents (Chiura et al. 2011). Not all horizontally transferred genes are retained, however, and ultimately selection pressures determine which acquired genes remain in the genome. Consequently, genes providing fitness advantages in the organism's ecological niche are more likely to be retained, while genes with negative fitness effects are rapidly purged and selectively neutral genes may be retained or lost through genetic drift (McInerney et al. 2017).

The diversity of relationships embedded in pangenomes allows us to view them as ecosystems (McInerney 2022). This ecological perspective helps understand that the natural emergence of epaktovars can take place through both deterministic and stochastic processes. In this ecosystem analogy, genes are viewed as “species” interacting within the genomic environment, while gene–gene relationships (mutualism, commensalism, competition) parallel species interactions in ecological communities. The acquisition and loss of genes resemble immigration and local extinction in island biogeography, and the core genome provides the foundational “habitat” upon which accessory genes establish themselves. This ecological framework can sometimes help explain why epaktovars emerge—the same “ecological rules” (expressed as likelihoods or probabilities) strongly influence gene acquisition and retention across diverse lineages, leading to convergent genome compositions (Dorrell et al. 2023). Even with the dynamic nature of HGT, there are predictable patterns that govern which genes will be acquired and retained together (Whelan et al. 2020).

Competition or gene–gene avoidance where a pair of genes appears to avoid being in the same genome, creates distinct xenotypes within a species, for example. Beavan et al. (2024) highlighted some examples from the *E. coli* pangenome that are clearly distinct xenotype groups. Strains containing the *pac* gene (involved in penicillin hydrolysis) form one xenotype group, while those containing the *symE* gene (a translational repressor) form another distinct xenotype group. These groups are not monophyletic, as judged by core gene phylogenetic analysis; instead they represent alternative evolutionary trajectories within *E. coli*, where genomes have acquired different horizontally transferred elements that appear incompatible within the same genome. In this case, we can say that, for the genomes sampled, the *pac* xenotype and the *symE* xenotype do not overlap in terms of membership.

Similarly, strains possessing the lgoT/mdtM transporters constitute one xenotype, while those carrying the nhaK/siaP/siaT/dctM:siaM transport systems form another and there is no overlap between the two, while their distribution overlaps extensively on a backbone phylogeny. These mutually exclusive xenotype patterns highlight how HGT can create distinctive genetic “types” within a species, with each xenotype potentially adapted to different ecological conditions or functional requirements that make the alternative gene sets incompatible.

Eukaryotes employ several mechanisms that parallel prokaryotic HGT while also employing unique processes that can create xenotypic relationships and epaktovar patterns. Interspecies hybridization, particularly common in plants and some animal lineages, can introduce large genetic repertoires from distantly related species (Abbott et al. 2013). Unlike the gene-by-gene transfer common in prokaryotes, hybridization can introduce thousands of genes simultaneously, creating dramatic xenotypic relationships. Additionally, transposable elements, which often comprise substantial portions of many eukaryotic genomes, serve as vectors for HGT across species boundaries and can reshape genome architecture (Schaack et al. 2010). The activity of these mobile genetic elements creates distinctive patterns of shared genetic material that fit our definition of xenotypes.

Endosymbiotic gene transfer represents another significant mechanism, where genes from organellar genomes (mitochondria and chloroplasts) or endosymbionts are incorporated into the nuclear genome (Timmis et al. 2004). This process has been particularly important in the evolution of plants, algae, and numerous protists, creating xenotypic relationships based on shared organellar genes. A particularly striking example of non-tree-like evolution comes from bdelloid rotifers, microscopic animals that have acquired approximately 8% of their genes from bacteria, fungi, and plants (Gladyshev et al. 2008; Nowell et al. 2024). These extensive horizontal acquisitions have created complex xenotypic relationships with diverse donors and potentially contributed to their remarkable survival in extreme environments.

### Case Studies of Epaktovars

The extensive study of *E. coli* genomes provides numerous examples of epaktovar formation. Different pathotypes of *E. coli* (EHEC, ExPEC, InPEC, ETEC, UPEC, etc.) have independently acquired similar virulence genes (Reid et al. 2000), leading to convergent pathogenic capabilities despite different evolutionary histories (Geurtsen et al. 2022). Additionally, different enteropathogenic bacteria (*Salmonella*, *E. coli*, *Shigella*) that have independently acquired type III secretion systems or similar toxin genes from different sources (Ogura et al. 2009). Different strains of pathogenic bacteria (e.g. the ESKAPE collection of pathogens for which new antimicrobial development is urgently needed) have independently acquired similar resistance gene cassettes through separate HGT events (De Oliveira et al. 2020), resulting in similar resistance profiles despite different genetic backgrounds. Distantly related bacteria living in extreme environments (like deep-sea vents (Moulana et al. 2020)) have independently acquired similar sets of genes for specialized metabolic pathways, such as sulfur oxidation (Ghosh and Dam 2009) or methane metabolism (Wiedenbeck and Cohan 2011), from different donor organisms. Some gut symbionts from different animal hosts are known to have independently acquired similar sets of genes for breaking down specific dietary components, resulting in similar metabolic capabilities despite different evolutionary histories (Wiedenbeck and Cohan 2011), while different endosymbionts of insects have independently acquired similar genes for nutrient provisioning, creating convergent genome compositions despite having different ancestral backgrounds (McCutcheon et al. 2009).

Eukaryotic systems provide compelling examples of epaktovar and xenotype patterns. There have been several transfers of β-carbonic anhydrase genes from prokaryotes to protozoans, insects, and nematodes (Zolfaghari Emameh et al. 2016) making these organisms xenotypes for these genes. Additionally, several lineages of herbivorous insects that are not each other's closest relatives have acquired a similar gene encoding a plant cell wall-degrading enzyme from microbial donors, enabling them to digest plant material through otherwise unavailable biochemical pathways (Wybouw et al. 2014). These insects are epaktovars with respect to their plant-digesting capabilities and xenotypes, due to the independent acquisition of the same homolog. In parasitic eukaryotes, convergent acquisition of similar molecular machinery for host invasion and immune evasion has been known to create epaktovar relationships. For instance, unrelated plant parasitic nematodes have independently acquired similar cell wall-degrading enzymes from bacterial donors (Haegeman et al. 2011).

Fungi exhibit a distinctive genomic architecture in which genes for specialized metabolic pathways are frequently physically clustered. Slot and Rokas (2011) identified a complete sterigmatocystin (ST) biosynthetic cluster spanning ∼54 kb and containing 23 genes in *Podospora anserina* that was horizontally acquired from *Aspergillus*. The transferred cluster maintains remarkable conservation in gene content, sequence similarity, microsynteny, and regulatory elements. Transcriptomic analysis confirmed expression of 14 cluster genes across multiple developmental stages, and independent verification of ST production in Podospora supports the functionality of this transferred pathway. This discovery of intact transfer of one of the largest known metabolic gene clusters combined with functional confirmation of the nature of the expression of the cluster makes *P. anserina* a xenotype and an epaktovar of the genus *Aspergillus*, despite these genera belonging to different taxonomic classes.

A more nuanced example of epaktovar formation involves marine actinomycete bacteria that produce iron-chelating compounds (siderophores). In the study by Bruns et al. (2018), all examined strains of *Salinispora tropica* and *Salinispora arenicola* possessed biosynthetic gene clusters (BGCs) for siderophore production, but these clusters were not all homologous. Two distinct types of siderophore BGCs were identified: one inherited vertically from the last common ancestor and another acquired through HGT, which in some cases displaced the ancestral cluster. As a result, all strains exhibit a similar phenotype—iron chelation—making them epaktovars. However, only the strains with horizontally acquired clusters are xenotypes with respect to this function.

The independent evolution of similar placental morphologies in mammals—driven by gains and losses of specific microRNAs—is an example of epaktovar formation. Although no genetic material is exchanged, the convergence arises through parallel changes in the same regulatory elements (miRNAs), leading to similar morphological outcomes (Fenn et al. 2025).

### Implications for Taxonomy and Classification

The prevalence of epaktovars and xenotypes in evolution challenges traditional taxonomic approaches that have been based solely on shared vertical ancestry (Yuan et al. 2023). When organisms independently acquire similar genes, they can appear more closely related than if gene acquisition has not taken place. Several approaches have been proposed to address this challenge including focusing on the stable core genome that is predominantly inherited vertically (Ciccarelli et al. 2006) and using the network-like structure of prokaryotic evolution through methods that visualize both vertical and horizontal relationships (Ragan et al. 2009). However, there is also the possibility of explicitly identifying epaktovars and xenotypes, by recognizing and annotating cases where similar genome compositions have arisen through independent acquisition. Importantly, the concepts of epaktovars and xenotypes do not invalidate hierarchical classification; rather, they enrich our understanding of the complex evolutionary processes that shape prokaryotic diversity.

Two distantly related organisms that share a particular homologous family and manifest the same phenotype can be considered xenotypes with respect to that family. Since this concept can be applied at several levels—promoter, protein domain, gene, operon, cluster, etc.—it is possible to define epaktovars for any evolving genomes, including plasmids, phages, and viruses. Indeed, mobile genetic elements like plasmids often exhibit xenotypic patterns as they acquire similar gene cassettes through independent horizontal transfer events.

These findings relate to Stephen J. Gould's “replaying the tape of life” thought experiment. While Gould suggested that evolutionary paths depend on unpredictable events (Gould 1989), research indicates that rewinding the evolutionary tape would still result in numerous predictable events (Beavan et al. 2024). The acquisition and retention of genes follows deterministic patterns influenced by gene–gene interactions and selection. This has profound implications for our understanding of evolutionary contingency versus determinism. While the specific sequence of evolutionary events may be contingent and unpredictable, the resulting patterns and structures show remarkable determinism and repeatability. Epaktovars and xenotypes are likely to emerge as a consequence of this deterministic component of evolution.

**Fig. 2.**
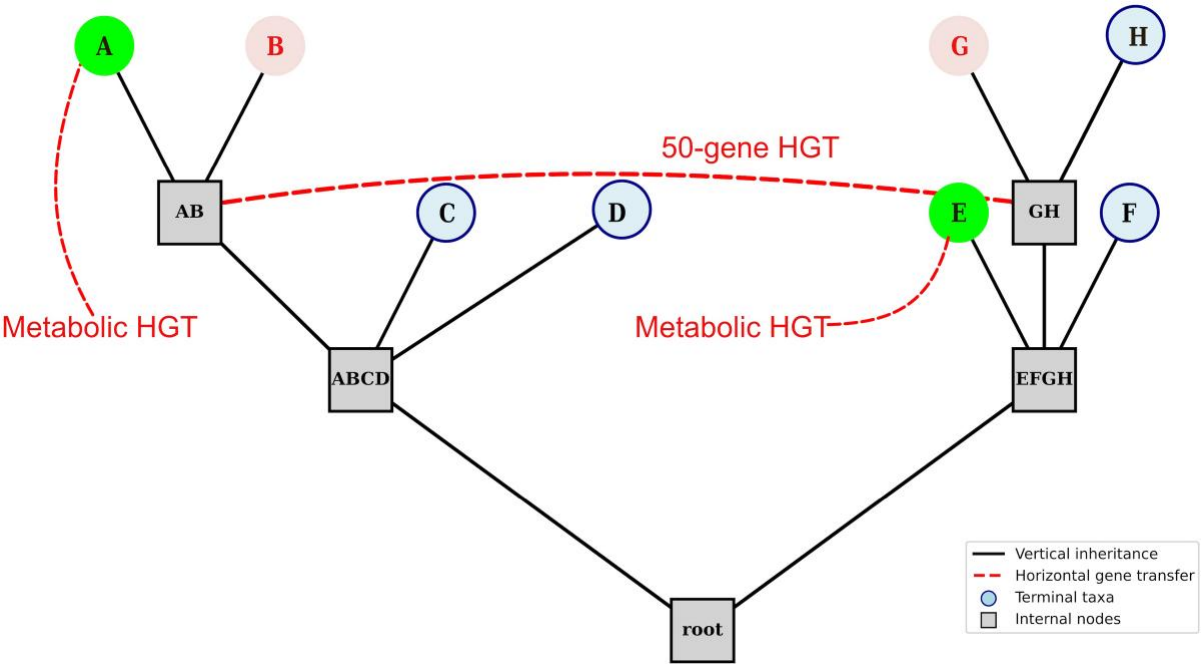
A phylogenetic network illustrating the relationships between epaktovars, xenotypes, and the consequences of vertical inheritance. Taxa {A,B} share 50 genes with taxa {G,H}, making them xenotypes of one another (see text for details). Without phenotypic data, we would not be able to say whether these groups are epaktovars. Taxa A and E, labeled green, are epaktovars and xenotypes for some trait, thanks to the independent acquisition of a gene encoding a metabolic enzyme, which results in the same phenotype in both taxa. B and G, labeled red, have independently evolved resistance to a toxin, though HGT has not facilitated the evolution of this trait, making them epaktovars, but not xenotypes.

To illustrate these concepts (see Fig. 2), consider eight hypothetical organisms (A–H), each with 1,000 gene families. In this dataset, five organisms (A–D) share 950 gene families not found in the others. These shared, derived genes are synapomorphic and support the interpretation of a monophyletic group formed through vertical inheritance. In addition to these, genomes A and B also share 50 gene families with G and H via HGT. These genes are absent in D and E, making A, B, G, and H xenotypes with respect to this horizontally acquired set. Now suppose taxa A and E, which are not closest relatives, independently acquire the ability to metabolize a particular substrate. If both acquired homologous genes via HGT, they would be xenotypes and epaktovars, but if they acquired different, non-homologous genes that produce similar functions, they would still be epaktovars, but not xenotypes. For contrast, imagine taxa B and G evolve resistance to a toxin via different mechanisms: B through point mutations in core genes and G through HGT from an unrelated species. Despite their distinct genetic routes, their shared phenotype makes them epaktovars.

These examples illustrate how different evolutionary relationships can coexist within the same set of organisms. Xenotypes focuses on the shared possession of genes thanks to the process of HGT regardless of their phenotypic effects, while epaktovars focuses on independently evolved similar phenotypes regardless of whether the underlying genetic mechanisms involve similar or different gene sets. The terminology helps distinguish between similarity due to common ancestry, similarity due to shared horizontally transferred genes, and similar phenotypes arising through independent acquisition events.

### Application to Synthetic Biology and De-extinction Efforts

The concepts of epaktovars and xenotypes have significant implications for synthetic biology and de-extinction efforts. Technological advances have made it easier to engineer organisms with novel gene combinations and to reconstruct the genomes of extinct species (Robert et al. 2017). These artificially created or modified organisms represent unique cases that challenge traditional taxonomic and evolutionary frameworks but can be effectively described using the terminology proposed here. Organisms that have been engineered to contain genetic material that evolved in other organisms are, by definition, xenotypes with respect to the donor organisms for those specific genes. For example, agricultural crops engineered to express bacterial Bt toxin genes (Perlak et al. 2001) share xenotypic relationships with the donor *Bacillus thuringiensis* strains, despite their vastly different evolutionary histories.

De-extinction efforts provide particularly contemporary examples. Recent advances in cloning technology have underpinned efforts to reconstruct extinct species such as the dire wolf, by inserting preserved DNA fragments into closely related modern species (Gedman et al. 2025). Consequently, these genetically modified organisms have complex evolutionary histories and relationships: they share genetic material with the extinct species they aim to resurrect, but they also contain genetic elements from the surrogate species used in the cloning process. They are therefore xenotypes with respect to both the extinct and surrogate species. Future de-extinction candidates such as woolly mammoths, dodos, or thylacines (Tasmanian tigers) would result in xenotypes of multiple donor organisms, if procured in this way. In addition, if these reconstructed organisms manifest phenotypic traits that are characteristic of their extinct counterparts—such as the cold-adapted physiology of mammoths or the distinctive coat patterns and hunting behaviors of thylacines—they would also qualify as epaktovars of the extinct species. This would be true even if the genetic mechanisms producing these phenotypes were not identical to those in the original extinct organisms, either because of technological limitations or intentional modifications for adaptation to contemporary environments.

Synthetic or engineering biology extends these concepts further through the creation of organisms with extensively engineered genomes (Shen et al. 2017; Goold et al. 2025). Microorganisms designed with synthetic metabolic pathways for bioremediation, biofuel production, or pharmaceutical synthesis often incorporate genetic elements from diverse donor species (Galdzicki et al. 2014; Paddon and Keasling 2014; Carbonell et al. 2018). These synthetic organisms form complex networks of xenotypic relationships with their various genetic donors and may qualify as epaktovars of naturally occurring organisms that have independently evolved similar metabolic capabilities.

The application of these concepts to synthetic biology and de-extinction highlights their usefulness beyond natural evolutionary processes. The line between natural and artificial evolution is becoming increasingly blurred, and consequently the terms epaktovar and xenotype are necessary to provide frameworks for understanding the complex relationships between engineered organisms and their natural counterparts while accommodating the unique processes by which these relationships are formed.

## Conclusion

The two concepts of epaktovars and xenotypes provide a framework for understanding how independently evolving lineages, whether prokaryotic, eukaryotic, plasmid, or viral, can acquire similar genomic patterns through gene transfer. Recent research demonstrates that despite the stochastic nature of evolution, gene content in bacterial pangenomes is significantly influenced by deterministic forces that repeatedly produce similar genomic configurations. By adopting the terms “epaktovar” and “xenotype,” we can describe and analyze more precisely the complex evolutionary relationships that characterize genomes and genomic elements such as phage or viruses. This terminology aligns with the existing concept of epaktologs at the protein level and provides a consistent framework for discussing genome similarity arising through HGT rather than common ancestry. Understanding these patterns has important implications for taxonomy, the prediction of evolutionary trajectories, and our conceptualization of evolution as a balance between contingency and determinism. As genomic data continue to accumulate, the identification and study of epaktovars will provide valuable insights into the rules governing gene acquisition, retention, and loss in evolving genomes.

## Data Availability

There is no new data associated with this manuscript.
